# Swimming-Induced Pulmonary Edema found in a U.S. Navy Basic Underwater Demolition/SEAL Recruit

**DOI:** 10.7759/cureus.29417

**Published:** 2022-09-21

**Authors:** Monica L Borza, Nicholas E Blonien

**Affiliations:** 1 General Physician, United States Navy, Pensacola, USA; 2 Occupational Medicine, HealthPartners Institute, St. Paul, USA

**Keywords:** navy seal, sipe, swimming, military personnel, cold-water immersion, pulmonary edema

## Abstract

A rare condition that can potentially be fatal, immersion pulmonary edema (also known as swimming-induced pulmonary edema, SIPE) occurs when the lungs fill with fluid during a physically exerting swim not associated with aspiration. This case study illustrates the diagnosis and treatment of swimming-induced pulmonary edema in a healthy young male recruit undergoing training at the United States Naval Special Warfare Basic Underwater Demolition/SEAL (BUD/S) course. This case report explores the clinical presentation, pathophysiology, and management of SIPE.

## Introduction

Swimming-induced pulmonary edema (SIPE) is a form of acute exertional pulmonary edema. Historically, exertional pulmonary edema is a well-known phenomenon described in racehorses, but it has rarely been described in humans [1]. It is estimated that approximately 40 cases of SIPE occur per year at the United States Naval Special Warfare Basic Underwater Demolition/SEAL (BUD/S) course. This calculates roughly to a 3% prevalence rate and has been shown to increase up to 5% in the colder months [2].

Classically, acute pulmonary edema can be categorized as either cardiogenic or non-cardiogenic. Non-cardiogenic acute pulmonary edema occurs when there is an increase in pulmonary capillary permeability. Acute pulmonary edema of cardiogenic origin occurs when the pulmonary capillary hydrostatic pressure exceeds the oncotic plasma pressure [3]. SIPE is a form of non-cardiogenic acute pulmonary edema usually found in the setting of cold-water immersion. The disease process is multifactorial and most likely attributed to physiological changes associated with water immersion and concurrent intense physical exertion [1]. Ultimately, these physiological changes, which include an increase in preload, afterload, and pulmonary vascular resistance, will lead to pulmonary capillary rupture and allow fluid to leak from the capillaries into the airspace [1].

Patients with SIPE classically present with dyspnea, cough, tachypnea, an altered mental status due to hypoxemia (oxygen saturation (SpO2) <92%), and occasional hemoptysis after a strenuous cold-water swim. The hallmark of SIPE has been described in the literature as a cough with a "frothy pink" sputum production [4]. SIPE usually improves rapidly with supportive care, and symptoms may completely resolve within 24 to 48 hours from the initial event.

In this case study, we will examine the rare clinical presentation of SIPE in an otherwise healthy 24-year-old U.S. Navy SEAL recruit during cold-water immersion training and explore its proposed pathophysiology.

## Case presentation

A healthy 24-year-old Navy SEAL recruit experienced sudden shortness of breath and productive cough immediately following a strenuous water immersion evolution during a particularly demanding BUD/S training week known as "Hell Week." The patient reportedly treaded for 20 minutes in seawater with a temperature of 14.4°C (59.7°F) immediately before the onset of symptoms. He was promptly removed from the water and assessed by supervising Navy medical personnel. The recruit was dyspneic and tachypneic, with a respiratory rate of 30 breaths per minute and a SpO2 of 88%. He was given supplemental oxygen via a non-rebreather mask and was transported to the on-site medical clinic for further evaluation.

**Figure 1:**
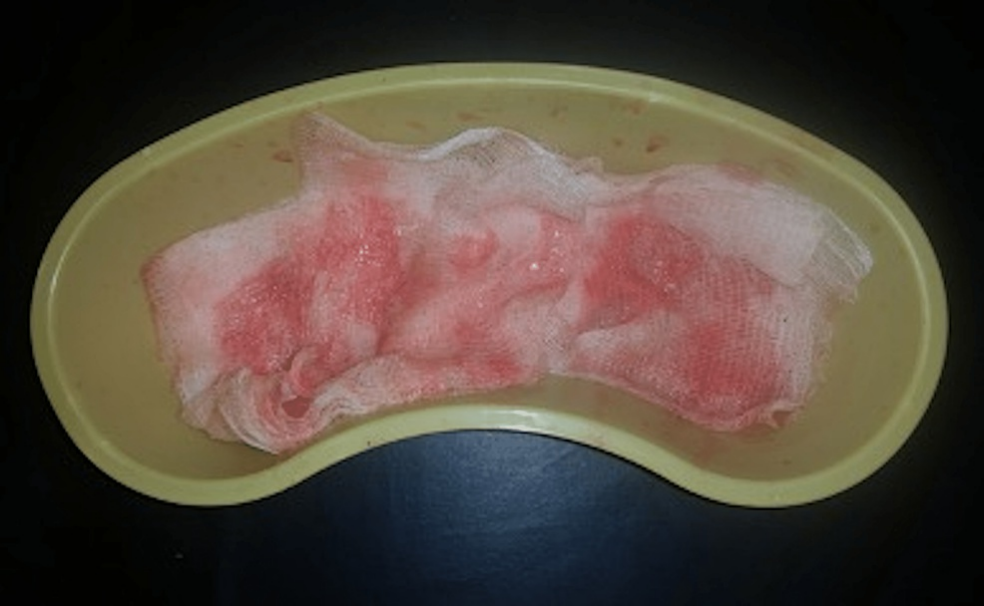
Frothy pink sputum coughed up by the patient.

Upon arrival at the clinic, the patient was shivering and continued to experience a cough of frothy pink sputum (Figure 1). He denied aspiration of seawater or whole-head immersion in water. He had no significant past medical history and denied any recent illness or infection. Before this event, the patient had completed three weeks of intense physical training without any injury and was found fit for full duty during a thorough medical screening before starting "Hell Week." This week is the most demanding period of BUD/S training, intentionally designed to push recruits to their mental and physical limits. Much of this week is spent in the Pacific Ocean, off the coast of southern California, where the candidates are cold, wet, and covered in sand for most of their training evaluations.

**Figure 2:**
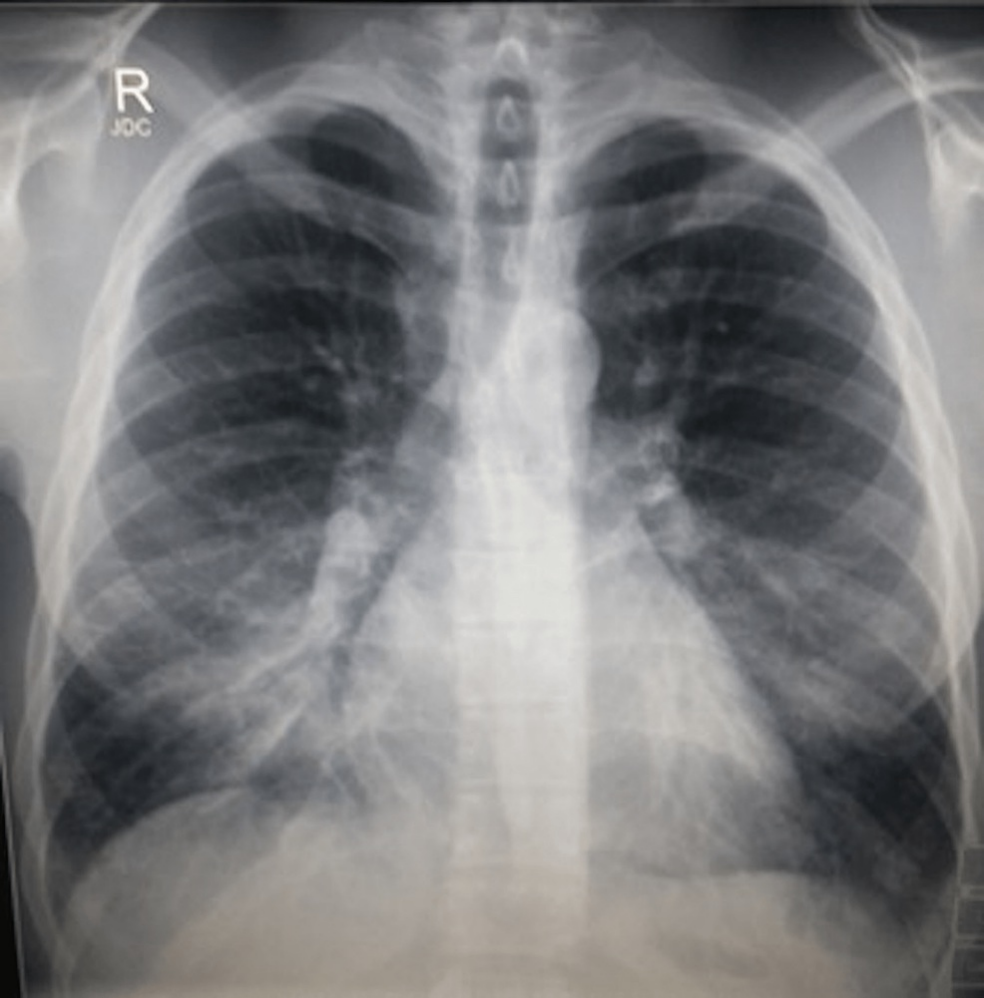
An upright chest radiograph with silhouetting of the right heart border and air bronchograms.

His vital signs were as follows: temperature of 35.3°C (95.5°F), blood pressure of 136/84 mmHg, pulse rate of 98 beats per minute, respiratory rate of 20 breaths per minute, and oxygen saturation of 91% on four liters of supplemental oxygen. A physical exam revealed rales and expiratory wheezes at the right lung base. The left lung field was clear to auscultation. The cardiovascular examination was routine, with no murmurs, rubs, or gallops. A chest radiograph showed silhouetting of the right heart border with air bronchograms (Figure 2). A diagnosis of SIPE was made based on history and objective findings.

The patient was treated with two courses of nebulized albuterol and supplemental oxygen via a non-rebreather mask at 4 L/min. He was wrapped in warm blankets, and a heating pad was placed behind his lower back for rewarming purposes. After completing the albuterol treatments, the patient underwent an exercise stress test on a stationary bicycle, requiring that he cycle for three minutes under moderate resistance at a speed of 90 revolutions per minute (RPM) while maintaining an SpO2 ≥95%, breathing room air. He passed the exercise stress test and was cleared to return to training. 

## Discussion

SIPE is of clinical relevance, particularly in the military community, and most common among Special Operations trainees. The pathogenesis of SIPE includes numerous biological, environmental, and exertional behavioral factors, but the exact mechanism of the disease process remains uncertain. A thorough literature review revealed that capillary stress failure, mainly due to exposure to relatively high transmural pressures, appears to be fundamental to the development of SIPE [1,5,6].

It has been postulated that intense exertional exercise can produce relatively high transmural pressures, ultimately leading to pulmonary edema [1,5,6]. Exertional pulmonary edema has been well studied in racehorses and causes increased pulmonary vascular pressure while galloping, which in turn causes stress failure of pulmonary capillaries [1]. Slade et al. suggested that increased cardiac output during average human exercise would rarely be expected to increase pulmonary capillary pressures to the point of capillary stress failure, except when combined with extreme exertion [7]. In this case report, a lung injury occurred during one week of exceptionally intense training. Operating on fewer than four total hours of sleep during this period, SEAL candidates are pushed to extreme physical and psychological limits. Roughly 25% of SEAL candidates complete ‘’Hell Week’’ and continue through the remainder of BUD/S training. Ludwig et al. performed a bronchoalveolar lavage on five BUD/S candidates diagnosed with SIPE. They found high levels of protein and red blood cells consistent with a capillary stress fracture, suggesting capillary rupture with exertion can occur in human beings [8].

The pathophysiological requirements for SIPE include a combination of intense physical exertion and physiological changes associated with water immersion. Head-above-water-immersion has cardiovascular as well as pulmonary effects. Water immersion increases venous return to the heart, leading to central blood pooling and increased cardiac preload. Immersion in cold water, in particular, is associated with a redistribution of blood flow from peripheral to thoracic vessels as a result of core body temperature decrease and peripheral vasoconstriction [1,4,5]. Arborelius et al. described head-above-water-immersion to increase cardiac output by 32% and increase mean pulmonary artery pressure [9]. Peripheral vasoconstriction causes central blood pooling, which raises cardiac preload and afterload and thus increases pulmonary vascular resistance [1,5,6]. SIPE results from a "perfect storm" of some combination of these physiological changes that overwhelms the human body’s ability to compensate, ultimately leading to pulmonary capillary leaking into the alveolar space. 

Patients with SIPE classically present with dyspnea, a cough that may be productive of frothy pink sputum, tachypnea, and occasionally altered mental status due to hypoxemia (SpO2 <92%) after an arduous cold-water swim. A significant hallmark of SIPE is a prompt resolution of symptoms within 24 to 48 hours of the initial insult. Because of this, conservative treatment is preferred and has emphasized supportive measures. To prevent further morbidity or mortality, it is imperative to provide supplemental oxygen to a patient with SIPE to maintain an oxygen saturation level above 94%. Other supportive measures include removing the patient from the cold-water environment, removing any wet clothing, and placing them in a warm climate. Beta2-agonists can be given for symptomatic relief and to promote alveolar fluid clearance. However, the role of pharmacotherapy has not been adequately studied.

Evidence has not yet demonstrated that individuals who have experienced an episode of SIPE are predisposed to additional re-occurrences. Shupak et al. described the only predisposing factor of significance as potentially low initial lung volumes [10].

## Conclusions

This case report highlights the potential for developing SIPE in young, healthy individuals, especially in military populations where exposure to extreme environments is commonplace. While physiological responses associated with cold-water immersion and intense physical exertion have been researched mainly as separate entities, combining these two stressors may result in pulmonary injury due to a poorly understood mechanism. Prompt recognition and management of SIPE are imperative for optimizing patient outcomes, especially those in high-risk training environments where extreme stressors are more commonplace. More research is warranted to understand this disease process and fine-tune supportive management fully.
